# The role of microRNAs in regulating inflammation and exercise-induced adaptations in rheumatoid arthritis

**DOI:** 10.1093/rap/rkac110

**Published:** 2023-01-24

**Authors:** Christopher Balchin, Ai Lyn Tan, Oliver J Wilson, Jim McKenna, Antonios Stavropoulos-Kalinoglou

**Affiliations:** Carnegie School of Sport, Leeds Beckett University, Leeds, UK; Leeds Institute of Rheumatic and Musculoskeletal Medicine, University of Leeds, Chapel Allerton Hospital, Leeds, UK; NIHR Leeds Biomedical Research Centre, Leeds Teaching Hospitals NHS Trust, Leeds, UK; Carnegie School of Sport, Leeds Beckett University, Leeds, UK; Carnegie School of Sport, Leeds Beckett University, Leeds, UK; Carnegie School of Sport, Leeds Beckett University, Leeds, UK

**Keywords:** inflammation, microRNAs, aerobic exercise, resistance exercise, physical activity, metabolism, disease activity, arthritis, risk factors, pathogenesis

## Abstract

MicroRNAs (miRNAs) are endogenously generated single-stranded RNAs that play crucial roles in numerous biological processes, such as cell development, proliferation, differentiation, metabolism and apoptosis. They negatively regulate target gene expression by repressing translation of messenger RNA into a functional protein. Several miRNAs have been implicated in the development and progression of RA. They are involved in inflammatory and immune processes and are associated with susceptibility to RA and disease activity. They are also considered to be potential markers of disease activity or even therapeutic targets. Likewise, several miRNAs are affected acutely by exercise and regulate exercise-related adaptations in the skeletal muscle and cardiovascular system and aerobic fitness. Interestingly, some miRNAs affected by exercise are also important in the context of RA. Investigating these might increase our understanding of the effects of exercise in RA and improve exercise prescription and, potentially, disease management. In this review, we focus on the miRNAs that are associated with both RA and exercise and discuss their roles in (and potential interactions between) RA and exercise-induced adaptations.

Key messages

- MicroRNAs are involved in the pathogenesis and progression of RA and in exercise-induced adaptations.
- Acute exercise changes levels of microRNAs commonly associated with disease progression in RA.
- Understanding the combined effects of exercise and RA on microRNA might help with personalizing exercise prescription and improve disease management.

## Introduction

RA is a chronic inflammatory autoimmune condition that primarily affects synovial joints. It is characterized by joint pain, stiffness and swelling, which can eventually lead to functional limitations and structural damage to the joints. People with RA tend to be physically inactive, with low levels of fitness and a high risk for cardiovascular and metabolic conditions.

Management of RA relies on pharmacological treatments aiming to reduce inflammation and its associated symptoms. In recent years, exercise has been included in the management recommendations for RA [1]. Well-designed exercise programmes are known to improve fitness, mobility, fatigue, overall health and quality of life among people with RA [2–4]. Importantly, exercise is now advocated as a non-pharmacological treatment for people with difficult-to-treat RA [5, 6] (i.e. people who remain symptomatic despite being treated based on existing pharmacological protocols [7]). However, relatively little is known about the biological regulation of these adaptations and how exercise and inflammation might interact in this respect in RA [8].

A relatively new area of investigation, particularly around the acute effects of exercise on bodily functions, is that of microRNAs (miRNAs). These are endogenously generated single-stranded RNAs that are ∼21–25 nucleotides in length [9]. More than 2000 miRNAs have been identified in humans [10], and they play crucial roles in numerous biological processes, such as cell development, proliferation, differentiation, metabolism and apoptosis [11]. They negatively regulate target gene expression by cleaving messenger RNA (mRNA) and subsequently repress translation of the mRNA into a functional protein [12]. Furthermore, they are estimated to contribute ∼1–2% of the whole genome and can regulate 30% of all protein-encoding genes [9]. Exercise-induced changes in miRNA levels are thought to regulate chronic adaptations in skeletal muscle, cardiovascular health and aerobic fitness [13, 14], and they might prove to be useful biomarkers for optimizing exercise prescription for the promotion of health or improvement of performance.

Moreover, miRNAs are suggested to play regulatory roles in inflammation and innate immune responses [15–17] and are essential in T cell activation during adaptive immunity [9]. Abnormalities in miRNA expression can contribute to RA pathology [18–21]. Dysregulation of miRNAs in peripheral blood mononuclear cells [22, 23], T lymphocytes [24], synovial tissue and synovial fibroblasts [18, 19] is associated with joint destruction, amplification of inflammation and degradation of extracellular matrix [25].

In this narrative review, we discuss some of the key miRNAs that have been linked to RA and explore the potential role of exercise in their regulation.

## MicroRNAs relevant to RA

A number of different miRNAs have been associated with RA susceptibility, disease progression and recurrence, in addition to drug response. Their study might reveal new therapeutic targets or biomarkers [26].

miR-16 is considered to regulate proliferation and differentiation of Th17 and Treg cells [27]. In normal conditions, miR-16 targets programmed cell death 4 gene (*PDCD4*) to suppress activation of inflammatory macrophages, which results in suppression of mRNA expression of pro-inflammatory cytokines TNF-α and IL-6 [28]. Indeed, miR-16 was shown to target the 3′ untranslated region of TNF-α [22, 29] and thereby, miR-16 might regulate TNF-α signalling [12], which is crucial for RA pathogenesis. Interestingly, miR-16 has been found at significantly lower levels in persons with early RA [30], but upregulated in established RA [30–32]. Nevertheless, miR-16 remains a reliable marker of disease activity in people with RA [12, 22].

miR-21 levels were also elevated in plasma of people with RA *vs* healthy adults [32]. It has been observed that miR-21 is expressed at higher levels in Treg *vs* Th17 cells. Also, signal transducer and activator of transcription 3 (*STAT3*), a transcription factor necessary for Th17 cell differentiation, is a target gene for miR-21 [33]. This contributes to an imbalance of Th17 and Treg cells, which highlights miR-21 as a biomarker of inflammation [33]. Additionally, there are considerations for miR-21 regulating apoptosis and mediating an anti-inflammatory response in macrophages [34, 35].

In plasma of people with RA, miR-24 levels were shown to be significantly higher in comparison to healthy individuals, while also correlating with disease activity (i.e. DAS28-CRP and DAS28-ESR) [36] and ACPA [12, 36]. ACPA is often (but not always) detected before the development of RA [37]. Therefore, in ACPA-negative people there could be some utility for assessing miRNA in RA diagnosis [12], because traditional techniques might not result in early diagnosis.

In healthy adults, miR-132 is activated by Th17 cells and enhances osteoclastogenesis by the downregulation of cyclooxygenase-2 transcription [38]. Conversely, in peripheral blood mononuclear cells from persons with RA, miR-132 expression levels are markedly higher, whereas concentrations of miR-132 in RA plasma are lower than in healthy individuals [22]. Interestingly, miR-132 levels were inversely correlated with tender joint count, and its role in systemic processes as a result of joint inflammation in RA has also been postulated [31].

miR-146a is one of the most extensively studied miRNAs in RA [12]. It has been shown to suppress nuclear factor kappa beta activity [39] and, in turn, suppress the inflammatory response [40–42]. Additionally, miR-146a targets TNF receptor-associated factor 6 (TRAF6) and IL-1 receptor-associated kinase 1 (IRAK1), two key molecules in Toll-like receptors and IL-1 signalling pathways [15]. However, in RA there is an absence of TRAF6 and IRAK1 regulation by miR-146a, which could contribute to the sustained production of TNF-α and thus amplify inflammation [22]. miR-146 expression is low in people with RA [30, 32] and is inversely correlated with CRP, ESR and TNF-α levels [12].

Expression of miR-155 is commonly increased in persons with RA *vs* healthy individuals [12, 43]. miR-155 is a potent regulator of the expression of cytokines [12], such as IL-1β, IL-6, IL-8 and TNF-α, while downregulating IL-10 production [44]. Subsequently, miR-155 expression in persons with RA has been positively correlated with IL-1β, TNF-α, CRP, ESR and DAS28 [45, 46]. Furthermore, miR-155 has a pleiotropic function and might regulate various signalling pathways related to the development of RA [19, 44, 45].

Decreased miR-221 expression is inversely associated with circulating levels of pro-inflammatory cytokines [47]. In RA, miR-221 expression is upregulated, leading to increased expression of VEGF, MMP-1 and MMP-3 [47], which are mediators of angiogenesis and inflammation [48]. Furthermore, overexpression of miR-221 could enhance RA synovial fibroblast activation and promote resistance to apoptosis [47].

miR-222 has identical seed regions, targets the same genes as miR-221 [49] and also affects angiogenesis and inflammation [50, 51]. Its expression increases with RA disease activity [51].

Previously, it was observed that miR-223 might regulate the differentiation of osteoclasts, which has implications for the joints in RA [52]. Subsequently, upregulation of miR-223 expression has been reported in people with RA [32]. High expression of miR-223 might contribute to severe synovitis and bone destruction [52]. Nevertheless, miR-223 expression does not appear to be correlated with DAS28, CRP or ACPAs in RA [21, 53], but circulating cell-free miR-223 might be a useful marker of disease activity in treatment-naïve persons with early RA [30].

## MicroRNAs and exercise response

Several studies have looked at the effects of exercise on a range of miRNAs. These are summarized below in Table 1.

**Table 1.** Studies investigating the effects of exercise on circulating microRNAs

Author	Participants	Exercise protocol	Time points of miRNA assessment	Changes in miRNA	Key findings and implications
Baggish *et al.* [54]	Healthy male competitive rowers (*n* = 14)	Cardiopulmonary exercise testing to determine maximum oxygen consumption, pre- and post-90-day training period. Incremental cycling using a stationary cycle ergometer	Baseline, immediately after and 1 h post-exercise	Pre-training: miR-21, miR-146a, miR-221 and miR-222 all increased (*P* < 0.05) post-exercise *vs* baseline Post-training: miR-146a and miR-222 increased (*P* < 0.05) Other miRNAs did not change post-exercise	Certain miRNAs were significantly upregulated after exhaustive aerobic exercise before and after exercise training
Baggish *et al.* [55]	Healthy male marathon runners (*n* = 21)	Marathon run	Baseline, immediately after and 24 h post-exercise	Immediately post-exercise: miR-1, miR-126, miR-133a, miR-134, miR-146a, miR-208a and miR-499 all increased (*P* < 0.05). The same miRNAs all decreased 24 h post-exercise to near baseline levels	miRNAs associated with inflammatory processes (i.e. miR-146a) were significantly upregulated immediately post-marathon Potential role for circulating miRNAs as unique markers of exercise physiology
de Gonzalo-Calvo *et al.* [56]	Healthy male amateur runners (*n* = 9)	10 km and marathon run	Baseline, immediately after, 24 and 72 h post-exercise	Immediately post-10 km run: miR-150 increased (*P* < 0.05) Immediately post-marathon: let-7d, let-7f-2, miR-125b, miR-132, miR-143, miR-148a, miR-223, miR-29a, miR-34a and miR-424 increased (all *P* < 0.05) Other miRNAs did not change post-exercise	Some inflammatory miRNAs responded to long-distance running in an exercise dose-dependent manner, whereas other miRNAs remained unchanged from baseline Exercise-induced inflammatory miRNA response parallels the classical inflammatory cascade (e.g. inflammatory cytokines)
Li *et al.* [58]	Healthy male basketball athletes (*n* = 10)	Cardiopulmonary exercise testing to determine peak oxygen consumption, using a stationary cycle ergometer	Baseline and immediately post-exercise	Immediately post-exercise: miR-21, miR-146a, miR-210 and miR-221 decreased (all *P* < 0.05) Other miRNAs did not change post-exercise	Potential role of circulating miRNAs reflecting the inflammatory responses post-acute exercise

CRF: cardiorespiratory fitness; hs-CRP: high sensitivity CRP; miR or miRNA: microRNA.

It appears that acute exercise impacts certain circulating miRNAs, and the response is dose dependent. However, the type of miRNA response to exercise as summarized in Table 1 varies significantly; for example, three studies identified that certain miRNA levels were upregulated immediately post-exercise [54–56], whereas two studies found that miRNA levels were downregulated at the same time point [57, 58]. Baggish *et al.* [54] examined the profiles of circulating miRNAs involved in various physiological processes, such as inflammation (miR-21 [59] and miR-146a [15]) and angiogenesis (miR-221 and miR-222 [60, 61]), both of which are associated with RA pathology. miRNA expression was measured at rest and after an acute bout of exhaustive cycling exercise in competitive male rowers before and after 90 days of aerobic training. Their findings demonstrated that certain circulating miRNAs were significantly upregulated after exhaustive exercise. The rapid upregulation of circulating miRNAs post-exercise could be explained by rapid increases in the cellular secretion or excretion of intracellular miRNA. Furthermore, miR-146a appears to alter the expression [62] of CD80 [63] and glucose transporter 3 [64], which are important to the inflammatory response and, subsequently, downregulated during acute exercise. Therefore, miR-146a could be involved in the anti-inflammatory processes exerted by exercise. Interestingly, they also observed a significant correlation between peak exercise miR-146a level and maximum oxygen consumption, which suggests that miR-146a could be a plasma-based marker of cardiorespiratory fitness.

In contrast, when Nielsen *et al.* [57] examined miRNA expression in response to acute exercise, they found that circulating miR-146a and miR-221 were downregulated immediately post-exercise, while there was no effect on miR-21 expression. The discrepancies could be explained by different acute exercise bouts, because participants in the study by Nielsen *et al.* [57] completed 1 h of cycling exercise at 65% of maximum power, whereas Baggish *et al.* [54] used a maximum oxygen consumption cycling protocol. Additionally, Nielsen *et al.* [57] used a different method when post-processing miRNA samples. Importantly, however, it appears that the observed increases in muscle-specific miRNAs were attributable to selective secretion rather than generalized passive release caused by exercise-induced muscle damage [57]. Li *et al.* [58] conducted peak oxygen consumption assessments using a cycle ergometer to investigate the miRNA response. They also found that certain miRNAs (i.e. miR-21, miR-146a and miR-221) were downregulated immediately post-exercise. The authors suggested that the decrease of these miRNAs might reflect the initial pro-inflammatory processes that accompany acute exercise, particularly at higher intensity.de Gonzalo-Calvo *et al.* [56] evaluated the response of circulating inflammatory miRNAs to different doses of acute aerobic exercise. Only miR-150 levels increased significantly after a 10 km race, whereas significant increases were observed in 12 miRNAs immediately after a marathon, with all levels returning to basal values 24 h post-race. The authors suggested that running a marathon is associated with major inflammatory stress, which might explain the increased expression of certain miRNAs. They also identified that inflammatory mediators such as IL-6, IL-8, IL-10 and hs-CRP all increased significantly after the marathon, and the circulating miRNA response post-exercise paralleled the inflammatory response. This indicated a dose-dependent effect of aerobic exercise on miRNA expression and systemic inflammation. Furthermore, the miRNA expression pattern observed after the marathon had predominantly anti-inflammatory effects, which might contribute to the exercise-induced anti-inflammatory response. An association has been found between miRNA activity and cytokine synthesis among healthy adults [65]. It has also been postulated that increased anti-inflammatory IL-6 levels post-exercise might be the result of increased miRNA activity, which implies a possible reciprocal relationship between miRNA and inflammation in healthy individuals [56]. However, this mechanism requires further investigation, particularly among people with RA.

In the only study to have looked at resistance exercise, Sawada *et al.* [66] recruited 12 males, who performed a resistance exercise session (consisting of bench press and leg press, five sets of 10 repetitions at 70% of one-repetition maximum). They found that 3 days after resistance exercise the miR-149 expression increased, whereas miR-146a and miR-221 expression decreased. A downregulation of circulating miR-146a and miR-221 contrasts with the findings from previous aerobic exercise studies [54, 55, 57]. Each exercise activates specific, and sometimes different, signalling pathways and subsets of genes transcriptionally regulated by miRNAs [67]. It is not clear whether circulating miRNAs are generated in skeletal muscle or other tissues post-exercise [66]. What is apparent is that post-exercise changes in miRNA expression can depend on the exercise mode, intensity and duration. Nevertheless, the collective findings indicate that miRNAs regulate several processes relevant to physiological exercise adaptations.

Importantly, no previous research has examined the impact of acute exercise on miRNA expression in RA, and only one study has investigated long-term effects of exercise on miRNAs. Özcan *et al.* [68] explored the effects of an exercise training programme in people with RA (*n* = 30) compared with a healthy control group (*n* = 30). People with RA completed strengthening and stretching exercises 2 days a week as part of an 8-week training programme. There was no difference between groups for miR-16 and miR-155 expression, and miR-146a expression was not affected by training.

## Summary and future directions

To summarize, miRNAs play a significant role in the development and progression of RA primarily by regulating the immune and inflammatory process. They might prove to be useful biomarkers of RA and help with early diagnosis, optimization of disease management and characterization of drug responses. This might prove important, particularly for people with difficult-to-treat RA. Nevertheless, there is a need to examine the downstream effects of miRNA changes on the expression of specific proteins, inflammation and disease characteristics inherent in RA. Furthermore, examining these effects in a population characterized by high-grade systemic inflammation might increase our understanding of the role of miRNAs in the regulation of biological processes even in the general population.

Exercise seems to affect miRNAs, but at the moment there is very little information in people with RA. However, there is limited understanding of the mechanistic role of miRNAs in exercise in people with RA. Furthermore, the evidence from the general population is conflicting, and no precise conclusions can be drawn on the specific mechanisms that miRNAs affect. Overall, miRNAs seem to regulate several of the adaptations induced by exercise, including muscle hypertrophy, cardiovascular fitness and angiogenesis. Exercise dose, but also individual variation, seem to affect their levels after an exercise session. Understanding the interaction of RA and exercise and their combined effects on miRNAs would allow for better planning of exercise programmes, evaluation of exercise-related benefits or risks and even optimization of disease management. Therefore, further research in the RA population investigating different exercises, doses and the role of miRNAs is required.

## Data Availability

No new data were generated in support of this manuscript.
